# Obesity Represses CYP2R1, the Vitamin D 25‐Hydroxylase, in the Liver and Extrahepatic Tissues

**DOI:** 10.1002/jbm4.10397

**Published:** 2020-08-26

**Authors:** Mahmoud‐Sobhy Elkhwanky, Outi Kummu, Terhi T Piltonen, Johanna Laru, Laure Morin‐Papunen, Maija Mutikainen, Pasi Tavi, Jukka Hakkola

**Affiliations:** ^1^ Research Unit of Biomedicine, Pharmacology and Toxicology University of Oulu Oulu Finland; ^2^ Biocenter Oulu University of Oulu Oulu Finland; ^3^ Medical Research Center Oulu Oulu University Hospital and University of Oulu Oulu Finland; ^4^ Department of Obstetrics and Gynecology, PEDEGO Research Unit, Medical Research Center Oulu University Hospital, University of Oulu Oulu Finland; ^5^ A.I. Virtanen Institute for Molecular Sciences University of Eastern Finland Kuopio Finland

**Keywords:** 25‐HYDROXYVITAMIN D, CYP24A1, CYP27B1, CYP2R1, HIGH‐FAT DIET, OBESITY, VITAMIN D RECEPTOR, VITAMIN D

## Abstract

Low plasma level of 25‐hydroxyvitamin D (25‐OH‐D), namely vitamin D deficiency, is associated with obesity and weight loss improves 25‐OH‐D status. However, the mechanism behind obesity‐induced vitamin D deficiency remains unclear. Here, we report that obesity suppresses the expression of cytochrome P450 (CYP) 2R1, the main vitamin D 25‐hydroxylase, in both mice and humans. In humans, weight loss induced by gastric bypass surgery increased the expression of CYP2R1 in the s.c. adipose tissue suggesting recovery after the obesity‐induced suppression. At the same time, CYP27B1, the vitamin D 1α‐hydroxylase, was repressed by the weight loss. In a mouse (C57BL/6N) model of diet‐induced obesity, the plasma 25‐OH‐D was decreased. In accordance, the CYP2R1 expression was strongly repressed in the liver. Moreover, obesity repressed the expression of CYP2R1 in several extrahepatic tissues, the kidney, brown adipose tissue, and testis, but not in the white adipose tissue. Obesity had a similar effect in both male and female mice. In mice, obesity repressed expression of the vitamin D receptor in brown adipose tissue. Obesity also upregulated the expression of the vitamin D receptor and CYP24A1 in the s.c. adipose tissue of a subset of mice; however, no effect was observed in the human s.c. adipose tissue. In summary, we show that obesity affects CYP2R1 expression both in the mouse and human tissues. We suggest that in mouse the CYP2R1 repression in the liver plays an important role in obesity‐induced vitamin D deficiency. Currently, it is unclear whether the CYP2R1 downregulation in extrahepatic tissues could contribute to the obesity‐induced low plasma 25‐OH‐D, however, this phenomenon may affect at least the local 25‐OH‐D concentrations. © 2020 The Authors. *JBMR Plus* published by Wiley Periodicals LLC on behalf of American Society for Bone and Mineral Research.

## Introduction

Vitamin D is a well‐established endocrine regulator of calcium homeostasis and bone mineralization. Moreover, vitamin D has multiple pleiotropic functions in extraskeletal target tissues.^(^ ^1^ ^)^ The consequences of vitamin D deficiency include poor bone development and health, as well as increased risk for many chronic conditions, such as cardiovascular disease, cancer, and autoimmune diseases.^(^ ^2^ ^)^ In epidemiological studies, vitamin D deficiency, reduced plasma 25‐hydroxyvitamin D (25‐OH‐D), is consistently observed in obese individuals,^3^, ^4^, ^5^ ^)^ and there is a strong, negative correlation between obesity and plasma 25‐OH‐D.^(^ ^6^ ^)^

Vitamin D is a prohormone activated in two enzymatic steps and begins with 25‐hydroxylation in the liver. Although several cytochrome P450 (CYP) enzymes are able to catalyze vitamin D 25‐hydroxylation in vitro, strong evidence indicates that CYP2R1 is the most important vitamin D 25‐hydroxylase in vivo.^(^ ^7^, ^8^ ^)^ Genetic defects in the CYP2R1 gene cause an inherited form of vitamin D deficiency and rickets in children.^(^ ^9^, ^10^ ^)^ Furthermore, large‐scale studies have identified CYP2R1 gene variants as one of the major genetic determinants of low 25‐OH‐D levels.^(^ ^11^, ^12^ ^)^ In the second bioactivation step, 1α‐hydroxylation is catalyzed by CYP27B1 in the kidney to produce the major active vitamin D receptor (VDR) ligand, namely 1α,25‐dihydroxyvitamin D [1α,25‐(OH)_2_‐D]. Interestingly, 25‐OH‐D itself may also have a weak agonistic effect on VDR.^(^ ^13^ ^)^ Additionally, the CYP24A1 is the main vitamin D catabolic enzyme which hydroxylates both 25‐OH‐D and 1α,25‐(OH)_2_‐D at the C24 position to render them inactive.^(^ ^14^ ^)^

Vitamin D 25‐hydroxylation has been considered a constitutive, unregulated step until recently.^(^ ^15^ ^)^ Based on this, the production of 25‐OH‐D has been considered to reflect the substrate's availability—the global supply of vitamin D. Therefore, the body's vitamin D status is usually assessed by measuring the circulating 25‐OH‐D level. Recently, we demonstrated that vitamin D bioactivation by CYP2R1 in the liver is tightly regulated by the metabolic state.^(^ ^16^ ^)^ CYP2R1 was repressed in mouse livers by fasting, obesity, type 2 diabetes, and type 1 diabetes and was associated with the downregulation of vitamin D 25‐hydroxylation activity.^(^ ^16^ ^)^ Similarly, Roizen and colleagues reported that obesity downregulates CYP2R1 and vitamin D 25‐hydroxylation in the mouse liver.^(^ ^17^ ^)^ These results indicate that, in contrast to previous assumptions, CYP2R1 expression and consequent vitamin D 25‐hydroxylation are actively regulated and may contribute to the changes in circulating 25‐OH‐D levels. Currently, there is no information whether energy homeostasis affects CYP2R1 regulation in humans or whether similar regulation occurs in extrahepatic tissues that express CYP2R1.

In the current study, we utilized adipose tissue samples from morbidly obese patients pre‐ and postgastric bypass surgery to show that obesity also affects CYP2R1 expression in humans. To further study the effect of obesity on vitamin D metabolism in more detail, we utilized a mouse model of high‐fat‐diet– (HFD‐) induced obesity, which is commonly used to study obesity, insulin resistance, and type 2 diabetes. Furthermore, we show that in mice, obesity affects CYP2R1 expression not only in liver, but also in several extrahepatic tissues.

## Materials and Methods

### Human subjects

Four female patients, aged 30 to 43 years, who underwent Roux‐en‐Y gastric bypass surgery at the Oulu University Hospital (Oulu, Finland), were enrolled in the study between 2017 and 2019. Surgical biopsy samples of abdominal s.c. adipose tissue under the umbilicus were obtained before and 11 to 19 months after bariatric surgery and snap‐frozen in liquid nitrogen. The characteristics of the study subjects are summarized in Table 2. The study involved all the patients who gave permission for the two biopsies during the collection period. All participants provided informed consent, and the study was approved by the Ethics Committee of the Northern Ostrobothnia District (Oulu, Finland; decision number 265/2016).

### Animal experiments

All animal procedures were approved by the National Animal Experimental Board, Finland (License numbers ESAVI/6357/04.10.07/2014 and ESAVI/8240/04.10.07/2017) according to the European Union directive 2010/63/EU. The investigators were not blinded during allocation, animal handling, and end point measurements. Mice were housed in individual cages under standard conditions with a 12‐hour dark–light cycle. The C57BL/6N mouse strain was used unless otherwise stated. Mice were obtained from the Laboratory Animal Center, University of Oulu. At the end of the experiments, mice were euthanized by CO_2_ inhalation and neck dislocation, blood was drawn into EDTA‐primed syringe from the vena cava, and tissues collected and snap‐frozen in liquid nitrogen.

#### *High‐fat–diet treatment*

Male and female mice aged 5 to 6 weeks were randomly allocated to a HFD (60% fat, Envigo TD.06414; Envigo, Huntingdon, UK; *n* = 7/group) or regular chow diet (Envigo 2018 Teklad Global 18% Protein Rodent Diet; *n* = 5/group) for 16 weeks. There was no significant difference in the mean weight between groups at the beginning. One male mouse in the chow group was euthanized before the completion of the study because of animal welfare reasons, and thus was omitted from the original study group. Immediately after the mice were euthanized, blood was taken from the vena cava, and the blood glucose levels were measured using Abbott FreeStyle Lite Blood Glucose Meter (Abbott Oy; Abbott Diabetes Care, Espoo, Finland). The daily intake of vitamin D was estimated based on the vitamin D content in the diets and the average amount of food consumption during the study. The mice in both the chow and HFD diets consumed approximately 0.16‐μg vitamin D/day.

#### Fasting experiment

Male WT mice aged 8 to 10 weeks were fed or fasted for 12 hours (*n* = 10/group), after which the mice were euthanized and tissues collected. Mice had free access to drinking water.

*Pgc‐1α* ^*−/−*^ mice (*n* = 6/group),^(^ ^18^ ^)^ age 3 to 4 months, and *Pgc‐1α* ^*+/+*^ (*n* = 7/group) littermates in the C57BL/6J background were fed or fasted for 12 hours before being sacrificed.

#### Dexamethasone treatment

Male mice aged 8 to 10 weeks were either treated with vehicle (DMSO plus corn oil) or dexamethasone (DEXA; Sigma‐Aldrich, St. Louis, MO, USA; *n* = 7/group) i.p. 3 mg/kg for 6 hours. The mice were fed *ad libitum* during the treatment period. Before the DEXA injection, some mice were injected twice (12‐hour time interval) with the glucocorticoid receptor (GR) antagonist mifepristone (MIF; Sigma‐Aldrich; MIF = 7, DEXA + MIF = 6) 50 mg/kg i.p. to antagonize the effect of the DEXA.

### RNA preparation and quantitative RT‐PCR

**Fig 1:**
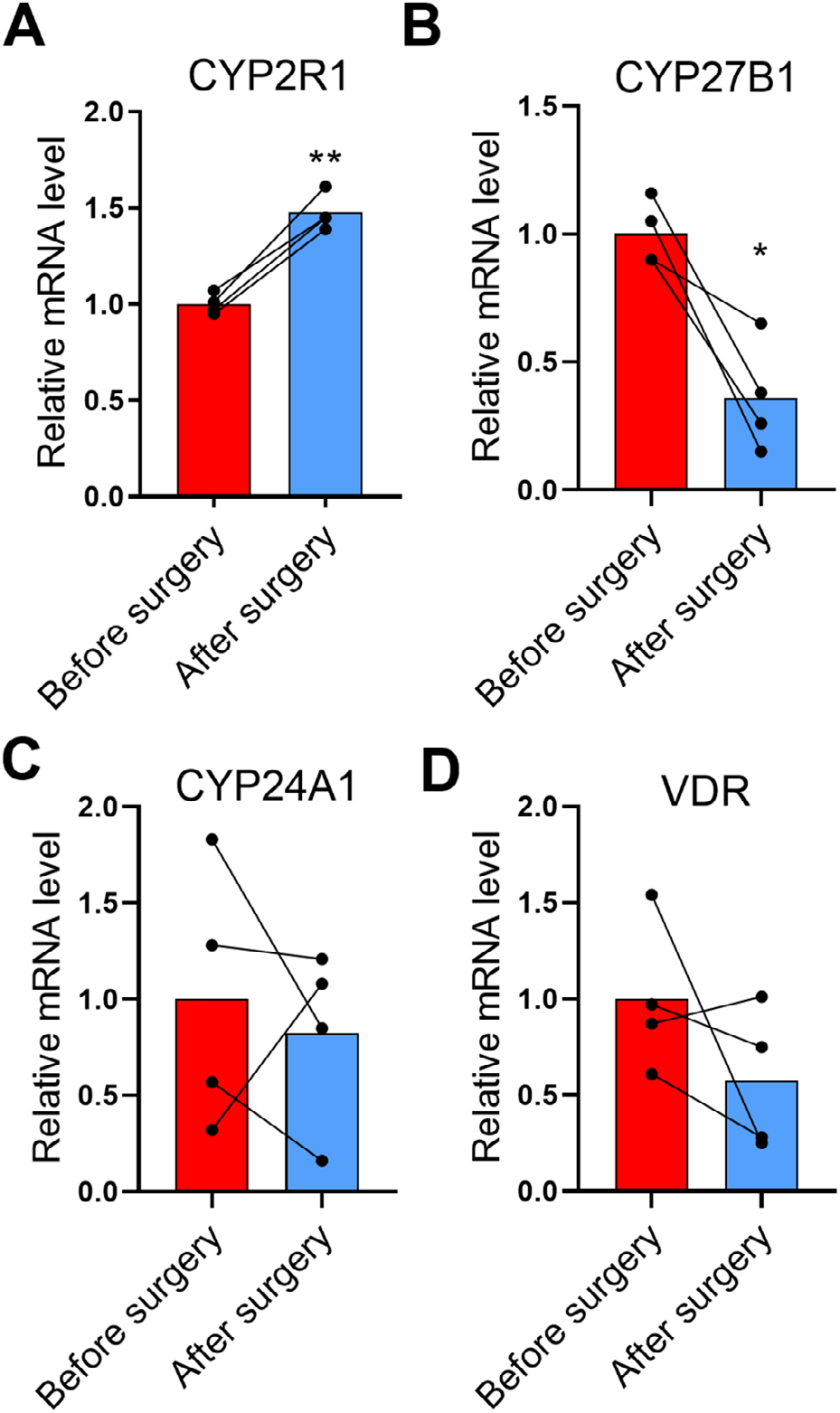
Effect of obesity surgery‐induced weight loss on vitamin D metabolism‐related mRNAs in human s.c. white adipose tissue. (*A*) CYP2R1, (*B*) CYP27B1, (*C*) CYP24A1, and (*D*) vitamin D receptor (VDR; *n* = 4). The data were analyzed with paired *t* test. Please note that the expression level of CYP27B1 was very low and close to the detection level.

**Fig 2:**
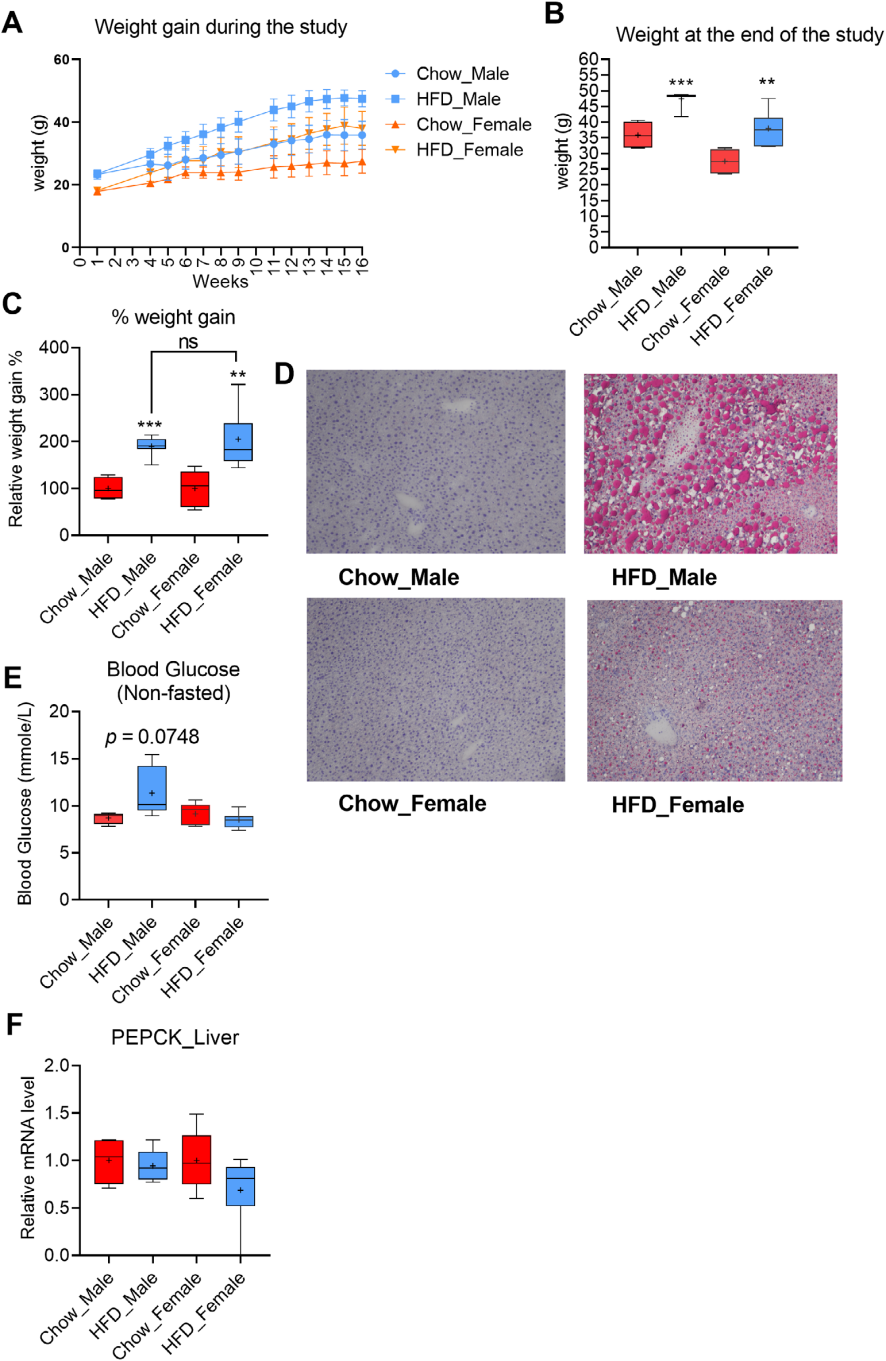
Effect of high‐fat diet (HFD) on male and female mice. (*A*–*C*) Weight gain in the male and female mice. (*D*) The oil red O staining of liver sections.10× magnification. (*E*) Nonfasted blood glucose. (*F*) PEPCK mRNA expression in the liver. Male, chow‐fed = 4, HFD = 7; female, chow‐fed = 5, HFD = 7. The box‐and‐whisker plots indicate the minimum, the 25th percentile, the median, the 75th percentile, and the maximum. In addition, the mean is indicated with +. All data were analyzed with a two‐tailed *t* test.

**Fig 3:**
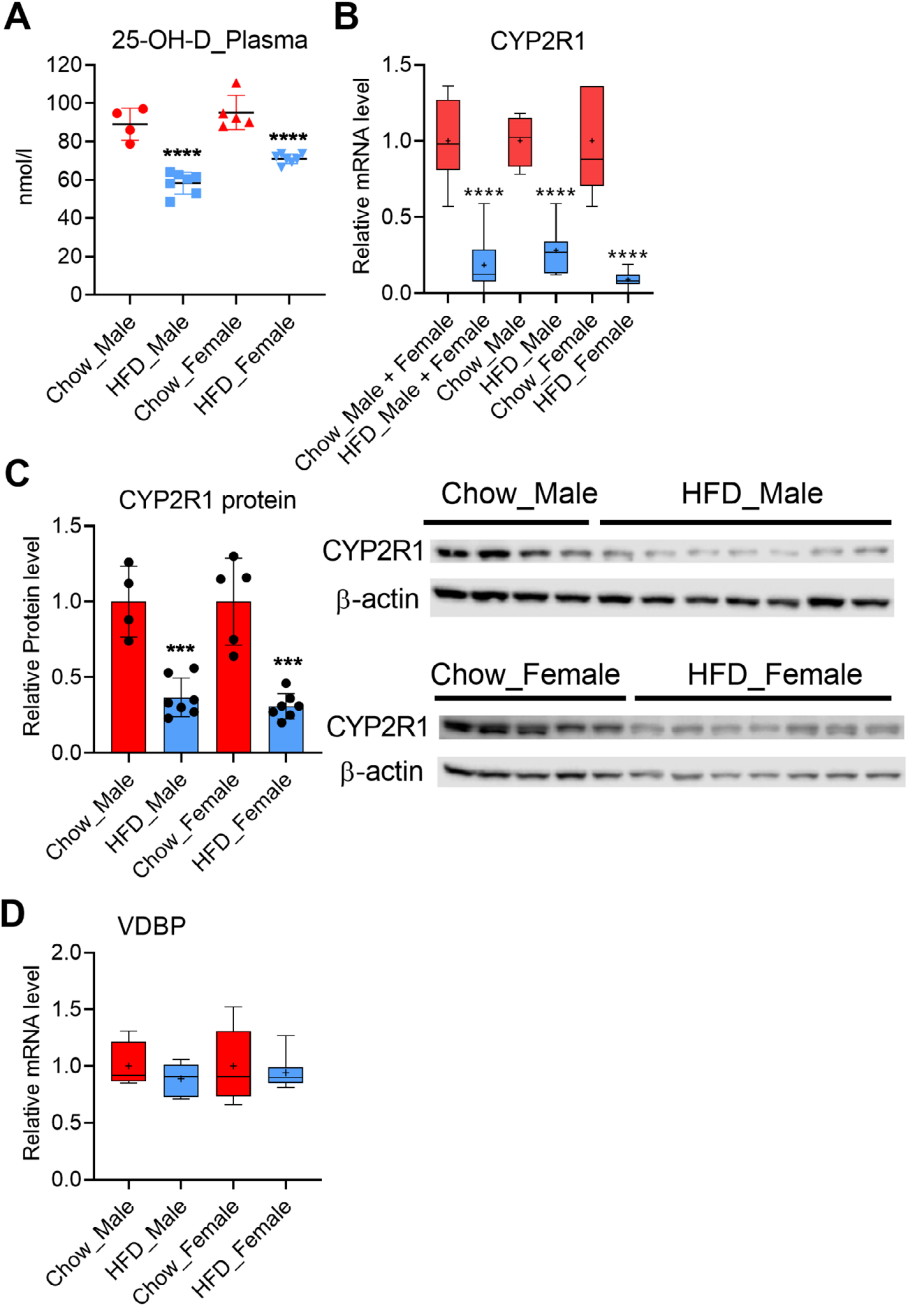
Obesity in mice reduces the plasma 25‐hydroxyvitamin D and represses the CYP2R1 in the liver. (*A*) Plasma levels of 25‐OH‐D. (*B*) Liver CYP2R1 mRNA expression in the males, females, and both genders combined. (*C*) Liver CYP2R1 protein expression. (*D*) Liver vitamin D binding protein mRNA. Male, chow‐fed = 4, HFD = 7; female, chow‐fed = 5, HFD = 7. The box‐and‐whisker plots indicate the minimum, the 25th percentile, the median, the 75th percentile, and the maximum. In addition, the mean is indicated with +. In the dot blot, the mean is indicated. All data were analyzed with a two‐tailed *t* test. HFD = high fat diet; VDBP = vitamin D binding protein.

Total RNA from the liver was extracted with RNAzol RT reagent (Sigma Aldrich, St. Louis, MO, USA) according to the manufacturer's protocol. One μg of RNA was used for cDNA synthesis with p(dN)6 random primers (Roche Diagnostics, Mannheim, Germany) using the RevertAid cDNA Synthesis kit according to manufacturer's protocol (Thermo Fisher Scientific, Waltham, MA, USA). The quantitative real‐time PCR reactions were conducted using SYBR green chemistry or TaqMan chemistry (Applied Biosystems, Foster City, CA, USA). The sequences for the primers and TaqMan probes are listed in Table 1. The fluorescence values of the qPCR products were corrected with the fluorescence signals of the passive reference dye (ROX, carboxy‐X‐rhodamine). The mRNA levels of target genes were normalized against TBP (TATA‐Box Binding Protein), 18S, or GAPDH reference genes using the comparative C_T_ (ΔΔC_T_) method as follow: Fig. 1*A* to *D* (GAPDH and TBP); Fig. 2*F*, 3*B*,*D*, 5*A* to *D*,*I*,*J*, 7*A* to *C*,*G* to *I*, 8*A* to *C*,*E* (TBP and 18S); Fig. 5*F* to *H* (18S); Fig. 6*A* to *C* (GAPDH, TBP, and 18S); Fig. 6 *D*, Fig. 7*D* to *F* (18S); Supplementary Fig. S2 (GAPDH and TBP); and Supplementary Figs. S3 and S4 (TBP and 18S).

**Table 1:** Sequences of the qPCR Primers

Gene	Forward primer (5′to 3′)	Reverse primer (5′to 3′)
*hCYP2R1*	GCCTCAGCTACCTCAGCATTT	CCATGAAAAGAATCGCCCACC
*hCYP27B1*	TCCATCCTGGGAAATGTGACA	ACAGGGTACAGTCTTAGCACTT
*hCYP24A1*	GATTTTCCGCATGAAGTTGGGT	CCTTCCACGGTTTGATCTCCA
*hVDR*	CCTTCAGGGATGGAGGCAAT	GCAGCCTTCACAGGTCATAGC
*hPPARγ*	CCACTATGGAGTTCATGCTTGTGAAGG	TGCAGCGGGGTGATGTGTTTGAACTTG
*hACACA*	AGTGGGTCACCCCATTGTT	TTCTAACAGGAGCTGGAGCC
*hUCP1*	AGGATCGGCCTCTACGACAC	GCCCAATGAATACTGCCACTC
*hUCP2*	CCCCGAAGCCTCTACAATGG	CTGAGCTTGGAATCGGACCTT
*hGAPDH*	GAGCGAGATCCCTCCAAAAT	GGCTGTTGTCATACTTCTCATGG
*hTBP*	CCCGAAACGCCGAATATAAT	ATCAGTGCCGTGGTTCGT
*mCYP27B1*	TCCTGGCTGAACTCTTCTGC	GGCAACGTAAACTGTGCGAA
*mCYP24A1*	CTGCCCCATTGACAAAAGGC	CTCACCGTCGGTCATCAGC
*mVDR*	GAATGTGCCTCGGATCTGTGG	GGTCATAGCGTTGAAGTGGAA
*mPPARγ*	CTCCAAGAATACCAAAGTGCGA	CCTGATGCTTTATCCCCACA
*mACACA*	CTTCCTGACAAACGAGTCTGG	CTGCCGAAACATCTCTGGGA
*mUCP1*	CCATCTGCATGGGATCA	GTCGTCCCTTTCCAAAGTG
*mUCP2*	ATGGTTGGTTTCAAGGCCACA	CGGTATCCAGAGGGAAAGTGAT
*mVDBP*	CCTGCTGGCCTTAGCCTTT	TGCTCAAATGTGCTACTGGAAA
*mPEPCK*	GGTGTTTACTGGGAAGGCATC	CAATAATGGGGCACTGGCTG
*m18S*	CGCCGCTAGAGGTGAAATTC	CCAGTCGGCATCGTTTATGG
*mTBP*	GAATATAATCCCAAGCGATTTG	CACACCATTTTTCCAGAACTG
*mGAPDH*	GGTCATCATCTCCGCCCC	TTCTCGTGGTTCACACCCATC
*mCYP2R1*	Mm01159414_m1 (Life Technologies)	

### The absolute quantification of the CYP2R1 using droplet digital PCR (ddPCR)

The CYP2R1 mRNA amount was quantified using droplet digital PCR (ddPCR) according to the manufacturer's protocol (Bio‐Rad, Hercules, CA, USA). The ddPCR workflow was conducted in three steps; generation of oil droplets, thermal cycling, and finally data acquisition and analysis. First, the PCR samples were prepared by pipetting 2‐μL cDNA (1/10 dilution), 10‐μL ddPCR supermix for probes, 1‐μL CYP2R1 Taqman mix (taqman probe and CYP2R1 primers), and 7‐μL water. After preparing all reaction mixtures, 20 μL of each reaction was loaded into a sample well of a DG8 cartridge (#186‐4008), followed by 70 μL of droplet‐generation oil for probes (#186‐3005) into the oil wells. For droplet generation, the cartridge was inserted into the QX200 droplet generator. After droplet generation, 40 μL of the droplet solution was transferred onto the PCR plate. The PCR plates were sealed with the PX1 PCR plate sealer. The two‐step PCR was conducted as follows: 95°C for 10 minutes followed by 40 cycles of 94°C for 30 seconds and 60°C for 1 minute. The last enzyme deactivation step was conducted at 98°C for 10 minutes. The PCR plates were kept at 4°C overnight. The PCR plate was inserted into the QX200 droplet reader to measure the positive and negative droplets. The CYP2R1 cDNA was quantified using the QuantSoft software.

### Western blot

CYP2R1 protein was detected from mouse liver and kidney microsomal fractions. Microsomes were extracted using differential centrifugation,^(19^ ^)^ and the protein was quantified by Bradford reagent (Bio‐Rad, Hercules, CA, USA). Protein fractions were separated using precast 10% SDS‐polyacrylamide gel electrophoresis. Proteins were then transferred into a nitrocellulose membrane (Millipore, Billerica, MA, USA) by a semidry method according to the manufacturer's protocol (Bio‐Rad). Membranes were blocked with 3% Amersham ECL Prime Blocking Reagent (GE Healthcare, Cardiff, UK) in Tris‐buffered saline for 1 hour. Then, the membranes were incubated with the anti‐CYP2R1 (center)‐Ab (SAB1300955; Sigma‐Aldrich; produced in Rabbit 1:500 in Tris‐buffered saline Tween‐20 [TBST]) overnight, followed by secondary horseradish peroxidase‐conjugated anti‐rabbit Ab (1:5000 in TBST) for 1 hour at room temperature.

The CYP2R1 protein bands were normalized against the reference protein β‐actin. Briefly, the membranes were stripped using mild stripping buffer (glycine buffer, pH 2.2), and were subsequently blocked again with 1% blocking agent in TBST for 1 hour. The membranes were incubated anti‐β‐actin‐Ab (produced in mouse, 1:5000 in TBST) overnight. Then, the membranes were incubated with secondary horseradish peroxidase‐conjugated anti‐mouse Ab (1:10000 in TBST) for 1 hour at room temperature.

The bands were visualized with Amersham ECL Start Western Blotting Detection Reagent (GE Healthcare) using Odyssey Fc (LI‐COR Biosciences GmbH, Bad Homburg vor der Höhe, Germany) and quantified by Image Studio software (LI‐COR Biosciences GmbH).

### Measurement of the plasma 25‐hydroxyvitamin D

The 25‐OH‐D levels were measured in the plasma using a Vitamin D^s^ EIA kit (Immunodiagnostic Systems Holdings Plc, Tyne & Wear, UK) according to the manufacturer's protocol.

### Statistical analysis

A Student's two‐tailed *t* test was used to compare the means of two groups, except for the mice depicted in Figure 7*A* (males only) and Figure 8*C*, the Mann–Whitney test was used. A paired *t* test was used to compare CYP2R1 expression before and 11 to 19 months after the gastric bypass surgery, normal disruption of the values was confirmed with the Shapiro–Wilk test. One‐way ANOVA followed by Tukey's post hoc test was used to compare multiple groups. All the statistical analysis was conducted using GraphPad Prism Software (La Jolla, CA, USA). Differences were considered significant at *p* < 0.05 (* *p* < 0.05; ** *p* < 0.01; *** *p* < 0.001; **** *p* < 0.0001).

## Results

### Weight loss after gastric bypass surgery upregulates CYP2R1 expression in human white adipose tissue

There is currently no information as to whether obesity affects CYP2R1 expression in humans. Moreover, it remains unknown how weight loss, promoted by gastric bypass surgery, affects vitamin D metabolizing enzymes. In the current study, we addressed these questions by utilizing adipose tissue samples from morbidly obese patients treated with gastric bypass obesity surgery. Subcutaneous white adipose tissue (WAT) biopsies were obtained from four obese female subjects before the surgical operation and 11 to 19 months after the surgery. During the study period, the patients lost 20% to 42% of their weight (Table 2).

**Table 2:** Characteristics of the Human Subjects Before and After the Surgery

Patient	Age (years)	Diseases	Weight (kg)		BMI (kg/m^2^)		Weight loss^a^ (%)	2nd Biopsy after the surgery (months)
			Before surgery	After surgery^a^	Before surgery	After surgery^a^		
2	43	Hypertension	117.4	77.8	49.8	33	−33.7	19
5	40	Type 2 diabetes, migraine, asthma	143.8	113.5	48.4	38.2	−21.1	13
6	30	None	129.4	75.4	46.4	27	−41.7	11
8	37	None	114.6	91.7	40.5	32.4	−20.0	12

a At the time of the 2nd biopsy.

CYP2R1 mRNA expression was significantly upregulated in adipose tissue samples taken after the gastric bypass surgery compared with the samples obtained before surgery. On average, the CYP2R1 was induced 1.5‐fold (*p* = 0.002) and the change was very similar in all patients (Fig. 1*A*). These results indicate that obesity affects CYP2R1 expression in humans and weight loss increases adipose tissue CYP2R1 expression. Analysis of the human tissue‐expression database (https://www.proteinatlas.org/)^(^ ^20^ ^)^ indicates that human CYP2R1 expression is ubiquitous in different tissues (Supplementary Fig. S1). Indeed, based on the qPCR Ct (Threshold cycle) values, the CYP2R1 expression can be estimated to be high in the adipose tissue samples studied.

Furthermore, we studied if weight loss in obese individuals affects the expression of the other major vitamin D metabolism enzymes and VDR in WAT. Interestingly, CYP27B1 expression was decreased by 64% (*p* = 0.02) after surgery (Fig. 1*B*) in all patients. In contrast to CYP2R1, CYP27B1 expression is relatively tissue selective and the expression is predominantly detected in the kidney (Supplementary Fig. S1). In accordance with this, CYP27B1 expression in the adipose tissue samples studied was close to the detection level.

In contrast, weight loss did not have any effect on CYP24A1 expression (Fig. 1*C*). There was a tendency for decrease in the VDR expression in three patients, but the effect was not significant (Fig. 1*D*). Furthermore, the genes under the regulation of VDR in the mouse adipose tissue,^(^ ^21^, ^22^ ^)^ Peroxisome proliferator activated receptor gamma (*PPARG)*, acetyl‐CoA carboxylase alpha (*ACACA)*, and uncoupling proteins (*UCP*) 1 and 2, were also measured, but none of them were consistently affected by weight loss (Supplementary Fig. S2).

### High‐fat diet induces similar weight gain, but more serious metabolic disturbance in the male mice compared with the female mice

A diet‐induced mouse model of obesity was used to further study the effect of obesity on vitamin D metabolism. The mouse obesity model, which was induced by a HFD, is commonly used to study obesity, insulin resistance, and type 2 diabetes. To investigate possible gender differences, both male and female mice were included in the study.

The mice were fed either with regular chow diet or HFD (60% fat) for 16 weeks. As expected, the mice on a HFD gained significantly more weight than the chow controls (Fig. 2*A*,*B*). The male mice displayed higher absolute weight gain than the female mice (11.6 and 10.5 g, respectively; Fig. 2*B*); however, the relative weight gain was similar for both genders (Fig. 2*C*).

The HFD feeding induced liver steatosis in both genders; however, the livers of the male mice were more steatotic than the female mice (Fig. 2*D*). The HFD‐fed male mice had a tendency for higher nonfasting blood glucose (11.3 ± 2.5 mmol/L) compared with the chow‐fed controls (8.7 ± 0.6 mmol/L; Fig. 2*E*), although there was no difference observed in female mice (Fig. 2*E*). Because fasting is known to have a marked effect on hepatic CYP2R1 expression,^(^ ^16^ ^)^ no fasting glucose was measured. Hepatic expression of the gluconeogenic gene PEPCK was not affected by a HFD in either gender (Fig. 2*F*). Based on the extent of liver steatosis and nonfasting blood glucose, the HFD induced a more serious metabolic disturbance in the male mice than the female mice, which corresponds with a previous report.^(^ ^23^ ^)^

### Diet‐induced obesity decreases plasma 25‐OH‐D and represses CYP2R1 expression in the mouse liver

When compared with the chow‐fed controls, obesity induced by a HFD reduced the plasma levels of the 25‐OH‐D by 35% and 25% in the male and female mice, respectively (Fig. 3*A*).

This corresponds with the expression of the CYP2R1, the major vitamin D 25‐hydroxylase in vivo, which was suppressed in the liver in the HFD group (Fig. 3*B*). A very efficient CYP2R1 repression was detected both in the male and the female mouse livers, 72% and 90%, respectively (Fig. 3*B*). Consistent with mRNA repression, the CYP2R1 protein level was also decreased in the livers of mice fed a HFD. Here, there was a significant downregulation in the male (63%, *p* = 0.0002), as well as in the female mouse (69%, *p* = 0.0001) hepatic CYP2R1 protein level (Fig. 3*C*).

Plasma 25‐OH‐D binds with vitamin D binding protein (VDBP), and VDBP plays a role as circulating reservoir for 25‐OH‐D. Thus, alterations in VDBP levels could affect the 25‐OH‐D plasma concentration. We therefore investigated the possibility that obesity could affect VDBP regulation. VDBP mRNA was measured in the livers of the mice fed a HFD and chow, but the HFD‐induced obesity had no effect on VDBP expression (Fig. 3D).

### Expression of CYP2R1 in the mouse extrahepatic tissues

Previous studies that describe CYP2R1 as the major microsomal vitamin D 25‐hydroxylase also reported that CYP2R1 was expressed the most in the testis, approximately 50% lower expression in the liver and much lower expression in the other mouse tissues studied.^(^ ^24^ ^)^ In contrast, according to the human tissue atlas,^(^ ^20^ ^)^ CYP2R1 appears to be expressed ubiquitously in the most tissues of humans (Supplementary Fig. S1).

**Fig 4:**
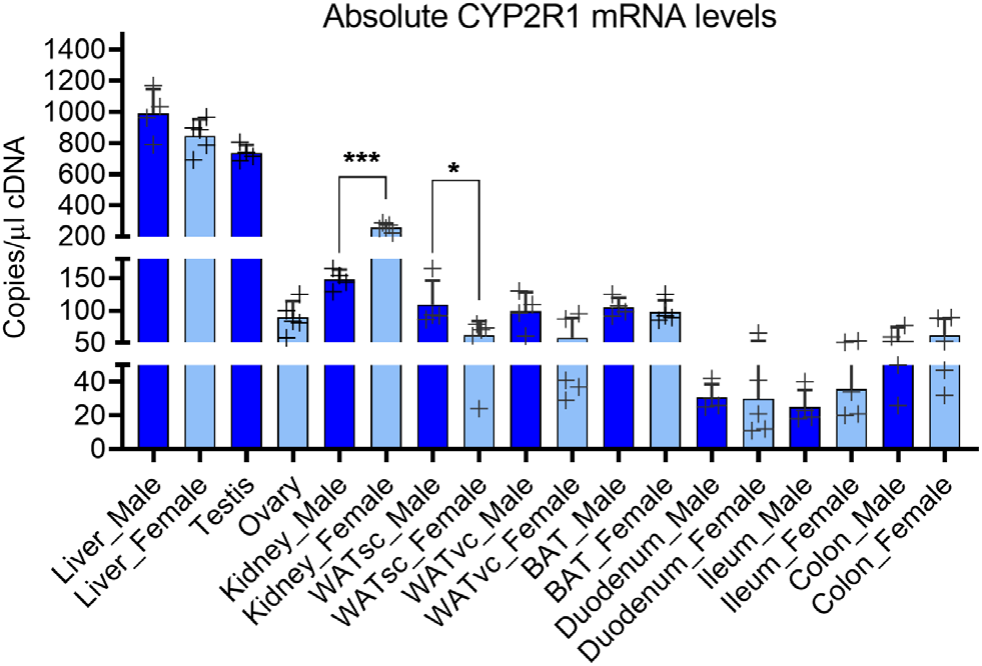
Absolute quantification of the CYP2R1 mRNA in liver and extrahepatic tissues of male and female mice. The bars indicate mean ± SD. Individual biological samples are indicated with +. The data were analyzed with a two‐tailed *t* test. BAT = Brown adipose tissue; WATsc = s.c. white adipose tissue; WATvc = visceral white adipose tissue.

To quantify the amount of the CYP2R1 mRNA expression in different mouse tissues, we performed ddPCR measurement. Here, CYP2R1 is broadly expressed and could be detected in all the tissues studied (Fig. 4). The highest expression of CYP2R1 was detected in the liver, and the level was similar in both males and females (Fig. 4). In males, the testis was the tissue with the second highest expression of CYP2R1 with relative expression level of 75% compared with the liver. Interestingly, among the tissues studied, CYP2R1 expression was the third highest in the kidney of males and the second highest in the females. The level of CYP2R1 mRNA was significantly higher in the female kidney than in the male kidney and CYP2R1 expression in the female kidney was 30% of that of the corresponding liver expression (Fig. 4).

Expression of CYP2R1 in adipose tissue was approximately 10% of that of the liver and the amount of CYP2R1 mRNA was comparable in all the fat deposits studied: s.c. (inguinal) and visceral (gonadal) WAT, as well as in the brown adipose tissue (BAT; Fig. 4). The lowest amount of the CYP2R1 was detected in the intestinal tract; namely the duodenum, ileum, and colon. However, among these, the colon had the highest amount of CYP2R1 (Fig. 4). There was also large interindividual variation in the expression in the intestinal tract. Altogether, these data revealed that the CYP2R1 is broadly expressed in mouse tissues, although the expression is the highest in the liver.

### Regulation of CYP2R1 expression by the obesity in the mouse extrahepatic tissues

**Fig 5:**
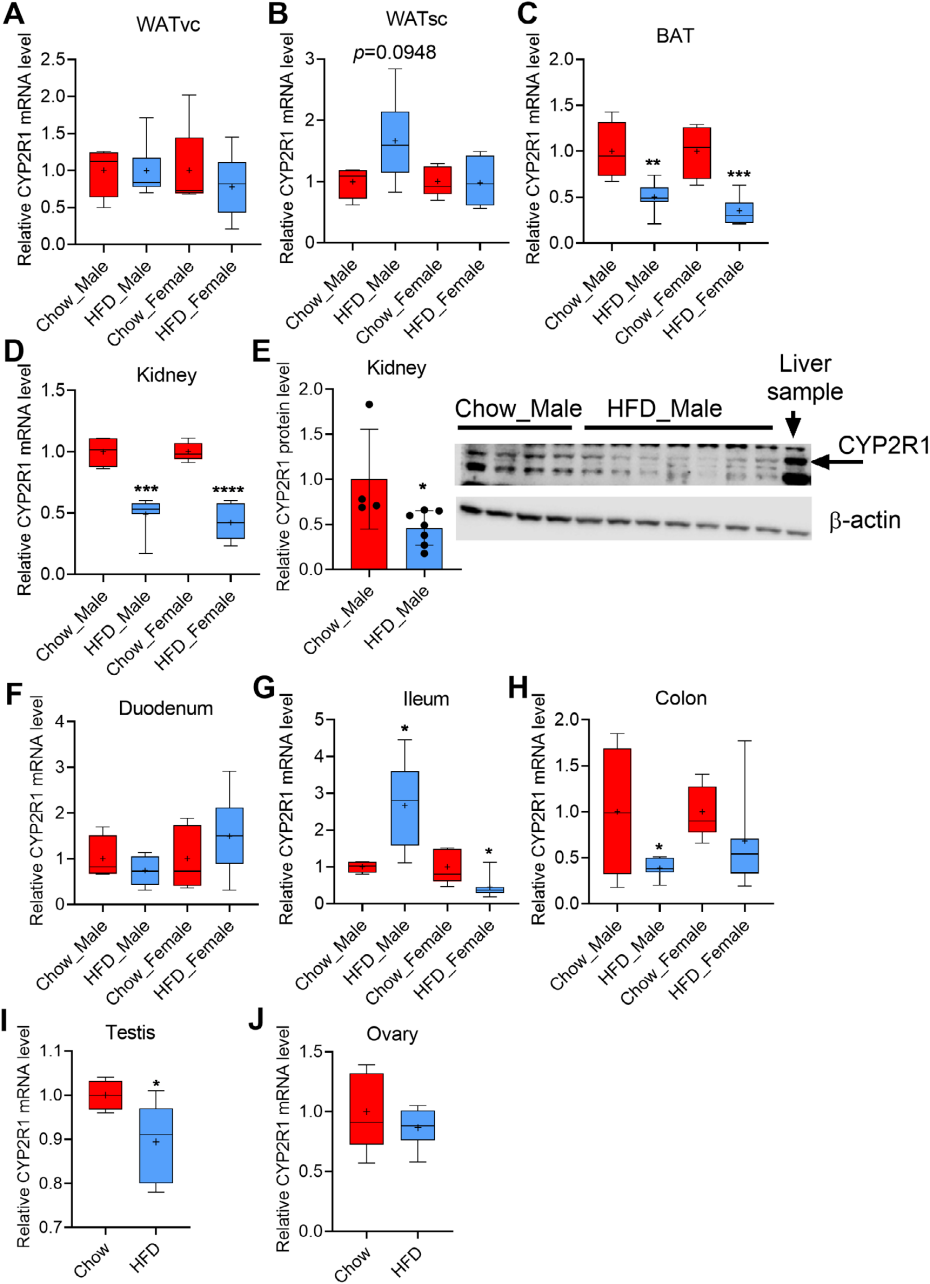
High‐fat‐diet– (HFD‐) induced obesity modulates the expression of CYP2R1 in extrahepatic tissues. Obesity had no significant effect on the CYP2R1 expression in (*A*) visceral white adipose tissue (WATvc) or (*B*) s.c. white adipose tissue (WATsc) in male and female mice. (*C*) Obesity repressed the CYP2R1 significantly in the brown adipose tissue (BAT). (*D*) Obesity significantly repressed the CYP2R1 mRNA in kidneys of male and female mice. (*E*) CYP2R1 protein was detected only in the male kidneys and it was decreased by obesity. (*F*) Obesity had no effect on the CYP2R1 expression in duodenum. (*G*) Obesity had an opposite effect on the CYP2R1 expression in the male and female ileum. (*H*) Obesity significantly repressed the CYP2R1 in the colon of the male mice. (*I*) Obesity repressed CYP2R1 in the testis, but (*J*) had no effect in the ovary. Male, chow‐fed = 4, HFD = 7; female, chow‐fed = 5, HFD = 7. The box‐and‐whisker plots indicate the minimum, the 25th percentile, the median, the 75th percentile, and the maximum. In addition, the mean is indicated with +. All data were analyzed with a two‐tailed *t* test.

We analyzed the effect of obesity on CYP2R1 expression in two separate WAT fat pads, s.c. (inguinal) and visceral (gonadal) WAT, and in the BAT. The HFD had no effect on CYP2R1 in the visceral WAT either in the male or the female mice compared with the chow‐fed controls (Fig. 5*A*). In the s.c. WAT, CYP2R1 expression was induced in some, but not all, of the male mice by HFD; the effect was not statistically significant (Fig. 5*B*). No effect of obesity induced by HFD was observed in the female s.c. WAT. Remarkably, obesity induced by a HFD strongly and significantly repressed the CYP2R1 mRNA expression in the BAT of both male and the female mice by 50% (*p* = 0.0066) and 65% (*p* = 0.0008), respectively (Fig. 5*C*).

Obesity induced by HFD significantly repressed the CYP2R1 mRNA levels in the kidneys of both the male and the female mice by 51%, and 58%, respectively (Fig. 5*D*). We next investigated if CYP2R1 repression could be observed at the protein level. Surprisingly, CYP2R1 protein was detected in the renal microsomes by immunoblotting only in the male mice and not in the female mice, despite the higher CYP2R1 mRNA expression in the female kidney (Fig. 5*E*). Consistent with the mRNA results, CYP2R1 protein was decreased in the kidney of the HFD‐fed male mice by 54% compared with the chow‐fed controls (Fig. 5*E*).

In the intestinal tract, we analyzed the CYP2R1 mRNA expression in several locations: the duodenum, ileum, and colon (Fig. 5*F* to H). Obesity induced by a HFD had no effect on CYP2R1 expression in the duodenum (Fig. 5*F*). Interestingly, in the ileum the HFD had the opposite effect on CYP2R1 in the male and female mice, increased in male and decreased expression in female mice (Fig. 5*G*). The HFD repressed CYP2R1 in both the male and female colon, however, with statistical significance only in male mice (Fig. 5*H*).

CYP2R1 mRNA expression is abundant in testis (Fig. 4). Interestingly, the HFD modestly, but significantly repressed CYP2R1 in the testis (Fig. 5*I*). The CYP2R1 was also found to be expressed in the ovary, but the basal expression was lower than in the testis (Fig. 4). A HFD had no effect on the CYP2R1 expression in the ovary (Fig. 5*J*).

### Fasting and activation of glucocorticoid receptor represses CYP2R1 in the kidney

**Fig 6:**
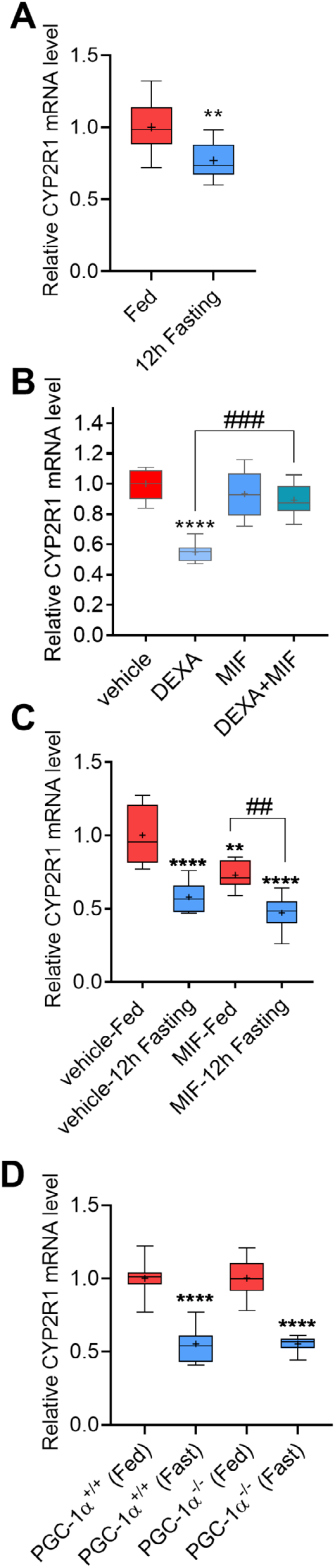
Fasting and activation of glucocorticoid receptor (GR) represses CYP2R1 in the kidney. (*A*) 12‐Hour fasting significantly repressed the CYP2R1 in kidney compared with chow‐fed controls (*n* = 10/group). (*B*) Dexamethasone (DEXA) treatment in mice significantly repressed the CYP2R1 in kidney, and cotreatment with the GR antagonist mifepristone (MIF) abolished the effect of DEXA (vehicle and DEXA, *n* = 7/group, MIF = 7, DEXA + MIF = 6). (*C*) GR antagonist MIF did not abolish the effect of fasting on the CYP2R1 in kidney (*n* = 8/group). (*D*) PGC‐1α knockout did not abolish the effect of fasting on the CYP2R1 in mouse kidney (PGC‐1α^+/+^ fed/fast *n* = 7/group, PGC‐1α^−/−^ fed/fast *n* = 6/group). The box‐and‐whisker plots indicate the minimum, the 25th percentile, the median, the 75th percentile, and the maximum. In addition, the mean is indicated with +. (A) Analyzed with two‐tailed *t* test. (*C*,*D*) Analyzed with one‐way ANOVA (Tukey's post hoc test, 95% CI). *significance compared to vehicle controls and significance compared as indicated in the figure.

Fasting and obesity involve activation of partially similar hormonal and signaling mechanisms. It has been demonstrated that the cortisol level is increased in obesity.^(^ ^25^ ^)^ Interestingly, 12‐hour fasting was found to repress CYP2R1 expression in the kidney by 23% compared with the chow‐fed control mice (Fig. 6*A*). Previously, we have shown that the glucocorticoid receptor (GR) and the coactivator peroxisome proliferator‐activated receptor gamma coactivator 1‐α/estrogen‐related receptor α (PGC‐1α‐ERRα) pathway play roles in the hepatic regulation of the *Cyp2r1* gene ^(^ ^16^ ^)^. Furthermore, in the kidney, CYP24A1 induction by fasting is mediated through the PGC‐1α‐ERRα pathway.^(^ ^16^ ^)^ Based on these findings, we hypothesized that GR and PGC‐1α could also play roles in the regulation of *Cyp2r1* gene in the kidney.

To characterize the putative role of GR, mice were treated with a synthetic glucocorticoid DEXA for 6 hours, and CYP2R1 expression in the kidney was analyzed. Interestingly, the DEXA treatment significantly repressed CYP2R1 expression by 45% compared with the vehicle control (Fig. 6*B*). To verify the involvement of GR in this repression, the mice were cotreated with DEXA and a GR antagonist MIF. MIF abolished the effect of DEXA on CYP2R1 repression, which indicates that GR indeed regulates the *Cyp2r1* gene in the kidney (Fig. 6*B*).

Next, the involvement of GR in the repression of the renal CYP2R1 by fasting was investigated. The mice were fasted for 12 hours and the effect of MIF on the fasting response was studied. Again, fasting significantly repressed CYP2R1 expression; however, MIF did not abolish the repressive effect of fasting (Fig. 6*C*). In addition, it was tested if the nutritional coactivator PGC‐1α could be involved in the regulation of the CYP2R1 in kidney, as it is a key player in the fasting response. To achieve this, PGC‐1α KO mouse model was used. Twelve‐hour fasting suppressed CYP2R1 expression equally in both WT and the PGC‐1α KO littermates (Fig. 6*D*). Thus, neither GR nor PGC‐1α appear to play a crucial role in the repression of CYP2R1 in the kidney by fasting.

### The HFD‐induced obesity has no effect on the CYP27B1 expression in the kidney

CYP27B1 is the sole 1α‐hydroxylase of 25‐OH‐D that produces the full active form of vitamin D, 1α,25(OH)_2_D. The kidney is the main site of CYP27B1 expression; however, some recent studies have reported expression also in extrarenal tissues, for example, in immune cells.^(^ ^26^ ^)^ Therefore, it was of interest to determine if HFD‐induced obesity could affect CYP27B1 expression in the kidney and other tissues. However, CYP27B1 mRNA was not detected in any of the tissues analyzed except the kidney, and obesity induced by a HFD had no effect on the renal CYP27B1 expression in either gender (Supplementary Fig. S3).

### The HFD‐induced obesity modulates the VDR expression in the tissues of male and female mice

The physiological function of vitamin D is predominantly mediated through VDR. VDR is widely expressed in most tissues. To further analyze the effect of obesity on the function of vitamin D, we analyzed the effect of HFD on VDR tissue expression. VDR was not detected in the liver, but it was abundant in all the extrahepatic tissues that were analyzed.

**Fig 7:**
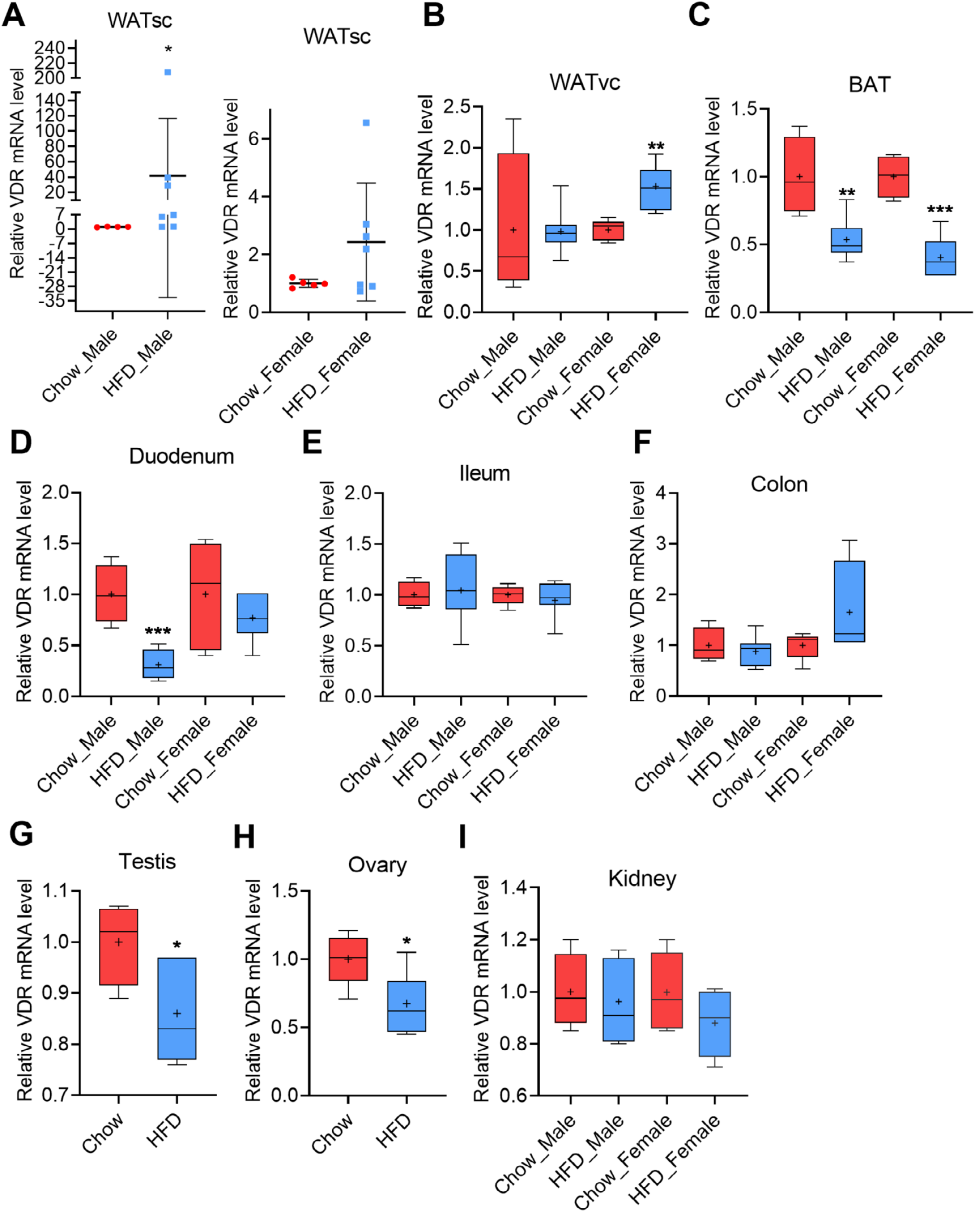
High‐fat‐diet– (HFD‐) induced obesity modulates the vitamin D receptor (VDR) expression in male and female mice tissues. (*A*) Obesity caused highly variable response in the s.c. white adipose tissue (WATsc) of male and female mice.(*B*) Obesity had no effect on the VDR expression in visceral white adipose tissue (WATvc) of male mice; however, it modestly induced VDR in the WATvc of female mice. (*C*) Obesity repressed VDR in the brown adipose tissue (BAT) in male and female mice. Obesity significantly repressed VDR expression in (*D*) duodenum of male mice only, whereas obesity had no effect on the VDR expression in (*E*) ileum and (*F*) colon. Obesity repressed the VDR in (*G*) testis and (*H*) ovary. (*I*) Obesity had no effect on the VDR expression in the kidneys. Male, chow‐fed = 4, HFD = 7; female, chow‐fed = 5, HFD = 7. The box‐and‐whisker plots indicate the minimum, the 25th percentile, the median, the 75th percentile, and the maximum. In addition, the mean is indicated with +. All data were analyzed with a two‐tailed *t* test, except (*A*) (males only) was analyzed with a Mann–Whitney test.

Interestingly, a HFD induced a highly variable response in the s.c. WAT of the male mice (Fig. 7*A*). A relatively high variation in response was also observed in the female mice, but in general the female mice displayed a more modest induction of VDR expression (Fig. 7*A*). VDR expression was not affected by HFD in the visceral fat of male mice; however, it was modestly, but significantly induced in female mice compared with the chow‐fed controls (Fig. 7*B*). In contrast, a HFD repressed VDR expression very clearly in the BAT of both male and female mice by 46% and 60%, respectively (Fig. 7*C*).

The intestinal tract is a well‐known VDR target tissue. In the duodenum of male mice fed a HFD, VDR was repressed significantly by 69% compared with the chow‐fed controls, but no effect was detected in the female mice (Fig. 7*D*). The HFD had no effect on the VDR expression in the ileum or colon of either gender (Fig. 7*E*,*F*).

Interestingly, obesity induced by HFD modestly, but significantly repressed VDR expression by 26% and 32% in the testis and the ovary, respectively (Fig. 7*G*,*H*). The VDR expression in the kidney was not affected by a HFD (Fig. 7*I*).

### Effect of HFD‐induced obesity on VDR target genes in the extrahepatic tissues

**Fig 8:**
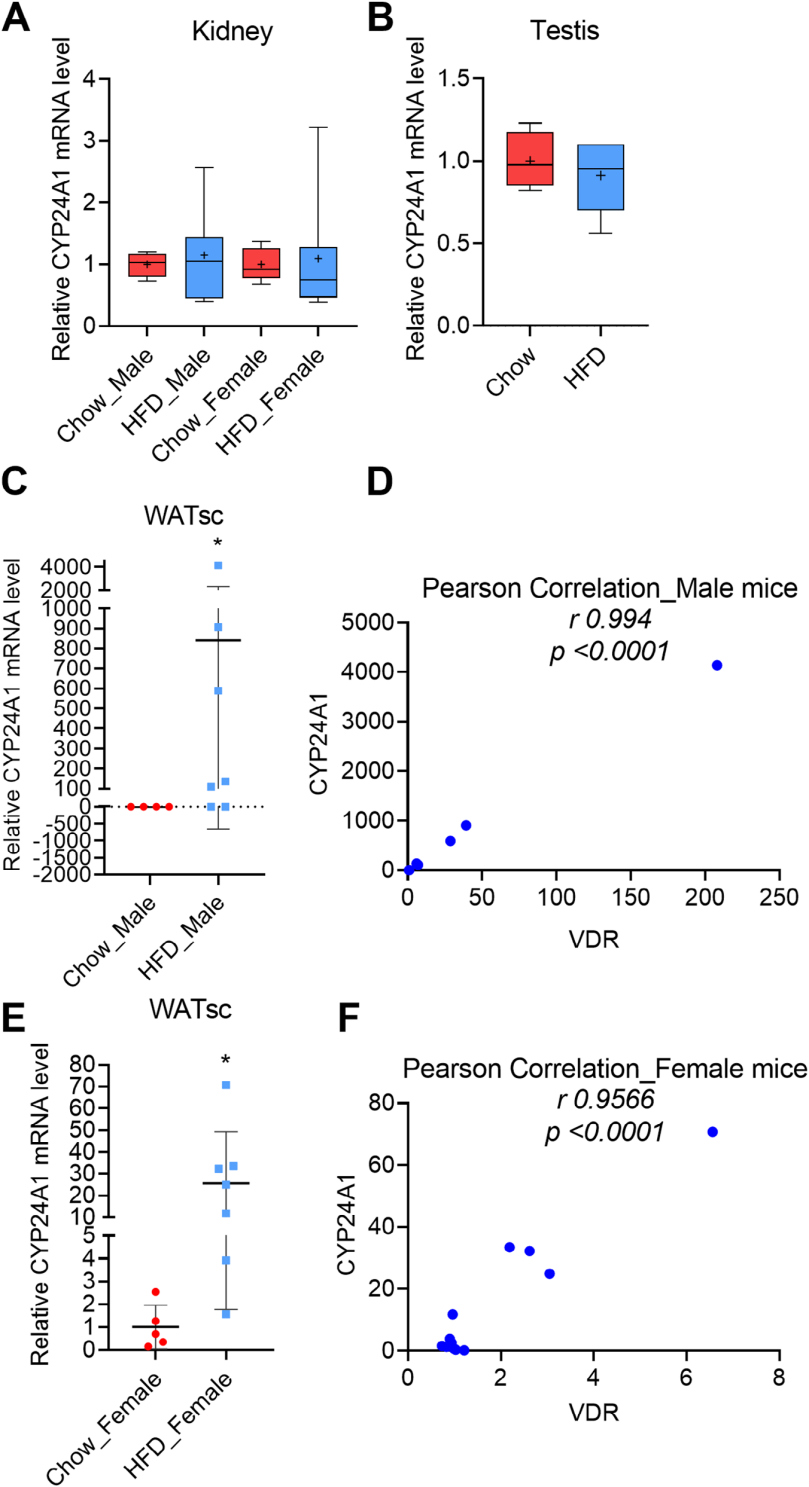
Effect of high‐fat‐diet– (HFD‐) induced obesity on CYP24A1 in the extrahepatic tissues. Obesity had no effect on the CYP24A1 expression in (*A*) kidney and (*B*) testis. (*C*) HFD caused highly variable response in the s.c. white adipose tissue (WATsc) of male mice. (*D*) Pearson correlation of CYP24A1 and VDR mRNA expression in the male mice. The analysis included both the chow‐ and HFD‐fed mice. (*E*) HFD caused variable response in the s.c. white adipose tissue (WATsc) of female mice. (F) Pearson correlation of CYP24A1 and vitamin D receptor mRNA expression in the female mice. The analysis included both the chow‐ and HFD‐fed mice. Male, chow‐fed = 4, HFD = 7; female, chow‐fed = 5, HFD = 7. The box‐and‐whisker plots indicate the minimum, the 25th percentile, the median, the 75th percentile, and the maximum. In addition, the mean is indicated with +. (*A*, *B*, *E*) were analyzed with the two‐tailed *t* test, and (C) was analyzed with a Mann–Whitney test.

CYP24A1 is efficiently regulated by vitamin D through VDR. Consequently, to assess the possible functional consequences of the modification of VDR expression by the HFD, CYP24A1 expression was also measured. In the kidney and testis, a HFD had no effect on CYP24A1 expression (Fig. 8*A*,*B*). Furthermore, in the intestine, ovary, and visceral fat, the CYP24A1 expression level was very low and could not be reliably detected.

Interestingly, CYP24A1 expression in the s.c. WAT was very low in the chow‐fed mice. However, there was a large interindividual variation in the response to a HFD in both genders, and in some male mice, there was a very efficient, up to several thousands of folds upregulation of CYP24A1 in response to a HFD (Fig. 8*C*,*E*). Because the variability in the CYP24A1 response to HFD resembled that of the VDR, a Pearson correlation analysis was done. Remarkably, there was a strong correlation between the CYP24A1 and VDR mRNA expression both in the male (*r* = 0.994, *p* < 0.001) and female (*r* = 0.9566, *p* < 0.0001; Fig. 8*D*,*F*) mice.

According to previous studies, expression of PPARG, ACACA, UCP1, and UCP2 are modulated in the adipose tissue by VDR knockout and overexpression.^(^ ^21^, ^22^ ^)^ Here, in BAT, ACACA expression was strongly suppressed and UCP1 expression induced by a HFD (Supplementary Fig. S4).

## Discussion

Human obesity is associated with vitamin D deficiency, and the epidemiological evidence suggests that a higher BMI leads to lower plasma 25‐OH‐D levels and not vice versa.^(^ ^27^ ^)^ There is consistent evidence that weight loss induced by diet restriction improves serum vitamin D concentration.^(^ ^28^, ^29^, ^30^, ^31^ ^)^ This obesity‐induced vitamin D deficiency can also be observed in mouse models. However, the mechanisms remain unclear. So far, there have been several explanations. For example, trapping of vitamin D in the large adipose tissue because of its hydrophobicity would reduce the available vitamin D for further hydroxylation by the liver and the kidney.^(^ ^32^ ^)^ Furthermore, volumetric dilution of vitamin D into the large body size in obese people has been proposed recently to be a key cause behind the lower plasma 25‐OH‐D levels.^(^ ^33^ ^)^ Additionally, low sun exposure caused by low mobility is another possible cause of lower vitamin D synthesis and vitamin D deficiency in obese subjects. Recently, we and others have reported that obesity represses CYP2R1 expression in the mouse liver, which results in lower vitamin D 25‐hydroxylase activity.^(^ ^16^, ^17^ ^)^ This suggests that reduced vitamin D bioactivation could play a role in obesity‐induced vitamin D deficiency. However, no human data are currently available.

In this study, we utilized samples from severely obese patients undergoing a gastric bypass operation. We were able to obtain s.c. fat samples from four patients before and 11 to 19 months after the operation; therefore, we could directly study the effect of weight loss on CYP2R1 expression in individual patients. The results were very similar and consistent in all patients: an approximately 1.5‐fold increase in CYP2R1 mRNA expression after weight loss. Although the study involved only four patients, follow‐up of the same patients before and after surgery greatly enhanced the reliability of the result. We therefore suggest that obesity represses CYP2R1 expression in human adipose tissue, and weight loss recovers the level of expression. Importantly, these data indicate that, similar to the mouse, CYP2R1 is also under the regulation of energy homeostasis in humans.

Liver is the main tissue in which vitamin D 25‐hydroxylation is catalyzed. Because of ethical reasons, it was not possible to get any liver samples from the patients; therefore, we are unsure if obesity also represses CYP2R1 in the human liver. Another limitation of this study is that the effect of the potentially altered 25‐hydroxylation on vitamin D status cannot be well‐studied in this model, as vitamin D deficiency is a common consequence of gastric bypass operation because of impaired gastric absorption.^(^ ^34^ ^)^

It is currently unclear whether obesity‐induced CYP2R1 repression in adipose tissue could contribute to the reduced plasma 25‐OH‐levels. Interestingly, according to the human tissue atlas,^20^ CYP2R1 appears to be expressed rather ubiquitously in most tissues, and the liver expression is not particularly high among human tissues (Supplementary Fig. S1).

Previous studies have indicated that in mice, CYP2R1 is predominantly expressed in the liver and testis and lower levels can be detected in several other tissues.^(^ ^24^ ^)^ We used ddPCR to more precisely analyze CYP2R1 tissue distribution in the mouse and showed that CYP2R1 is broadly expressed. However, the highest expression was detected in the liver and testis in accordance with previous results.^(^ ^24^ ^)^ This supports the results obtained from the rodent studies which show that the liver is a major site for vitamin D 25‐hydroxylation.^(^ ^35^ ^)^

There are obviously important differences in the tissue distribution of CYP2R1 between a human and a mouse, which may suggest that the human liver could play a less predominant role in vitamin D 25‐hydroxylation compared with the mouse. Interestingly, patients with unilateral orchiectomy as a result of testicular cancer have lower serum 25‐OH‐D levels,^(36^ ^)^ which suggests that in humans, other tissues besides liver could also contribute to the circulating 25‐OH‐D levels. Nevertheless, the extrahepatic tissues that express CYP2R1 may convert vitamin D into 25‐OH‐D locally, which may contribute to autocrine or paracrine functions of vitamin D in these tissues.^(^ ^37^ ^)^ However, according to our measurements, the extrarenal expression of CYP27B1 seems to be very limited in the tissues studied. Interestingly, it has been shown that the 25‐OH‐D itself could inhibit the sterol regulatory element‐binding protein, which is a key player in the regulation of lipid homeostasis.^(^ ^38^ ^)^

Consistent with previous results, we observed strong, obesity‐induced downregulation of CYP2R1 expression in mouse liver.^(^ ^16^, ^17^ ^)^ Although the HFD‐feeding induced more severe liver steatosis in the male mice than the female mice, it had a similar effect on CYP2R1 expression. Therefore, liver steatosis does not appear to play a major role in the repression of CYP2R1 as a result of obesity. Furthermore, there are no major gender differences in the CYP2R1 response to obesity.

VDBP is another key determinant of 25‐OH‐D levels, and polymorphisms in the *GC* gene (that codes for VDBP) are, along with the *CYP2R1* variants, among the key genetic causes for low 25‐OH‐D.^(^ ^11^, ^39^ ^)^ Lower VDBP production in the liver can result in lower 25‐OH‐D levels in plasma.^(^ ^39^ ^)^ However, unlike CYP2R1, we did not observe any effect of obesity on the VDBP expression in mouse liver, which indicates that these two key determinants of vitamin D status are regulated differently.

In addition to the liver, CYP2R1 expression was repressed in several extrahepatic tissues in response to obesity. In the kidney and BAT, CYP2R1 was consistently repressed in both genders. Furthermore, CYP2R1 repression was observed in the testis. Among the tissues we studied, these were also the tissues with the highest CYP2R1 expression after the liver. One previous study reported upregulation of CYP2R1 in the gonadal fat of male mice after 11 weeks on a HFD.^(^ ^40^ ^)^ We did not observe a similar effect in our study using a 16‐week‐HFD treatment, and did not detect any significant effects of obesity on CYP2R1 expression in mouse WAT.

In our previous study, we demonstrated that, besides obesity, fasting also represses CYP2R1 in the liver, which involved potentially both the GR and PGC‐1α‐ERRα pathways.^(^ ^16^ ^)^ Furthermore, the PGC‐1α‐ERRα pathway induced CYP24A1 in the kidney in response to fasting. In the current study we observed that fasting also represses CYP2R1 expression in the kidney. Furthermore, GR activation by DEXA also repressed renal CYP2R1. However, neither GR nor PGC‐1α was indispensable for the fasting‐elicited repression of renal CYP2R1, which suggests that additional, unknown pathways may play a role. Further studies are required to clarify if either GR or the PGC‐1α‐ERRα pathway is involved in obesity‐induced repression of CYP2R1 in the kidney.

We studied the effect of obesity on VDR expression in different mouse tissues. The most clear and consistent finding both in males and females was the repression of VDR expression in BAT. This coincided with the upregulation of UCP1, a VDR target gene and the key regulator of adaptive thermogenesis. Indeed, VDR KO mice have induced UCP1 and display a lean phenotype.^(^ ^41^, ^42^ ^)^ Repression of both CYP2R1 and VDR, as well as associated upregulation of UCP1 in BAT, in response to a HFD could be hypothesized to be a counterregulatory mechanism of the body to fight against energy excess. However, the causality behind the gene responses cannot be assessed based on the current results. Obesity induced VDR and CYP24A1 expression in the s.c. adipose tissue of a subset of mice; however, no effect was observed in human s.c. adipose tissue.

In conclusion, we demonstrated that CYP2R1 expression is regulated by energy homeostasis both in humans and mice. In humans, weight loss induced by gastric bypass surgery increased the expression of the CYP2R1 in s.c. adipose tissue. We propose that this represents recovery after the obesity‐induced repression. In mice, obesity repressed CYP2R1 in the liver, kidney, BAT, and testis. Although CYP2R1 repression in mouse liver is likely to contribute to the obesity‐induced low plasma 25‐OH‐D concentrations, the systemic effect of repression in the extrahepatic tissues currently remains unclear. However, the substantial extrahepatic expression also suggests an important role for CYP2R1 outside the liver. This may be related to the local vitamin D effects.

## Disclosures

TP serves occasionally on advisory boards for Exeltis, Merck, and Ferring, and has lectured at educational events for Gedeon Richter, Ferring, Roche, Merck, and AstraZeneca. LM‐P has lectured at educational events for Merck, Ferring, Gedeon Richter, and Biomerieux. MM is currently an employee of the Charles River Discovery, Kuopio, Finland. JH has received royalties as an editor of a book from the Duodecim Publishing Company Ltd. All other authors declare no competing interests for this work.

## Author Contributions

**Mahmoud‐Sobhy Elkhwanky:** Conceptualization; formal analysis; investigation; writing‐original draft. **Outi Kummu:** Investigation; writing‐review and editing. **Terhi Piltonen:** Conceptualization; investigation; writing‐review and editing. **Johanna Laru:** Investigation; writing‐review and editing. **Laure Morin‐Papunen:** Conceptualization; writing‐review and editing. **Maija Mutikainen:** Investigation; writing‐review and editing. **Pasi Tavi:** Investigation; writing‐review and editing. **Jukka Hakkola:** Conceptualization; formal analysis; funding acquisition; supervision; writing‐original draft.

## Authors' roles

MSE, JH, LM‐P, and TP designed the study. JL and TP collected the human adipose tissue samples. MM and PT performed the PGC‐1α KO mouse experiments. MSE and OK performed all the other the animal experiments. MSE performed all measurements. MSE and JH analyzed the data and wrote the manuscript. All authors read and approved the final version.

### Peer Review

The peer review history for this article is available at https://publons.com/publon/10.1002/jbm4.10397.

## Supporting information


**Appendix S1**: Supplementary Information.

## Data Availability

All data related to the study are presented in the manuscript or in the Supplemental Information.
