# Supplementary material for: Obesity Represses CYP2R1, the Vitamin D 25‐Hydroxylase, in the Liver and Extrahepatic Tissues
Source: JBMR Plus. 2020 Aug 26;4(11):e10397. doi: 10.1002/jbm4.10397 (PMC7657391; doi:10.1002/jbm4.10397)
Supplement: Supplementary file 1 — Appendix S1: Supplementary Information. [file JBM4-4-e10397-s001.pdf]

Supplemental data

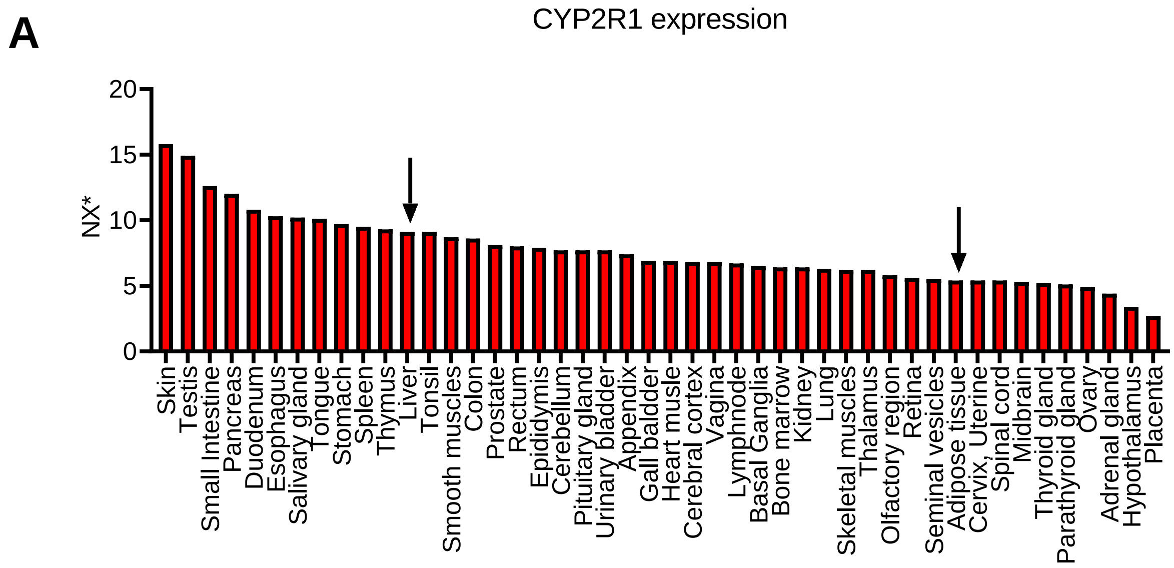

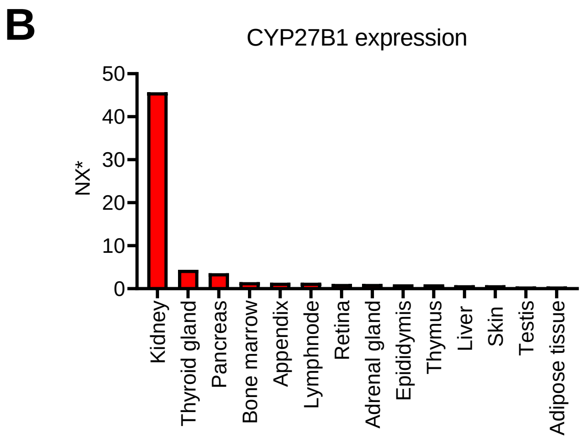

Supplemental Figure 1. Expression of CYP2R1 and CYP27B1 mRNA in the selected human tissues. All data were retrieved from the human protein atlas database. (https://www.proteinatlas.org/ENSG00000186104-CYP2R1/tissue) (https://www.proteinatlas.org/ENSG00000111012-CYP27B1/tissue)
*NX is abbreviated for normalized expression. For more information, please see: https://www.proteinatlas.org/about/assays+annotation#normalization_rna

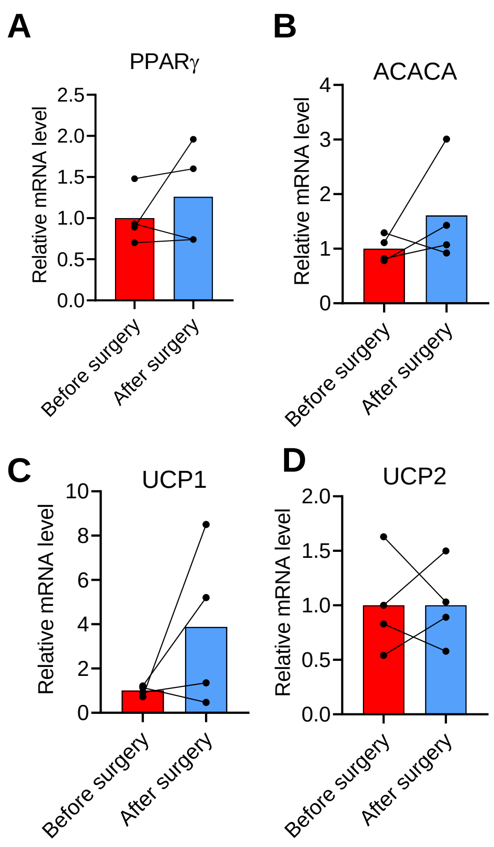

Supplemental Figure 2. Obesity in human and weight loss induced by gastric bypass surgery had no clear effect on the VDR target genes i.e., PPARg, ACACA, UCP1, and UCP2 in the subcutaneous white adipose tissue. All data were analyzed with paired t-test.

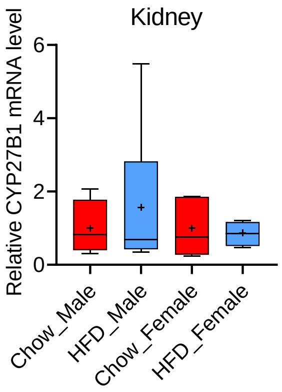

Supplemental Figure 3. HFD induced obesity had no effect on the CYP27B1 expression in kidneys of male and female mice. The box-and-whisker plot indicate the minimum, the 25th percentile, the median, the 75th percentile, and the maximum. In addition, the mean is indicated with +. The data was analyzed with the two-tailed t-test.

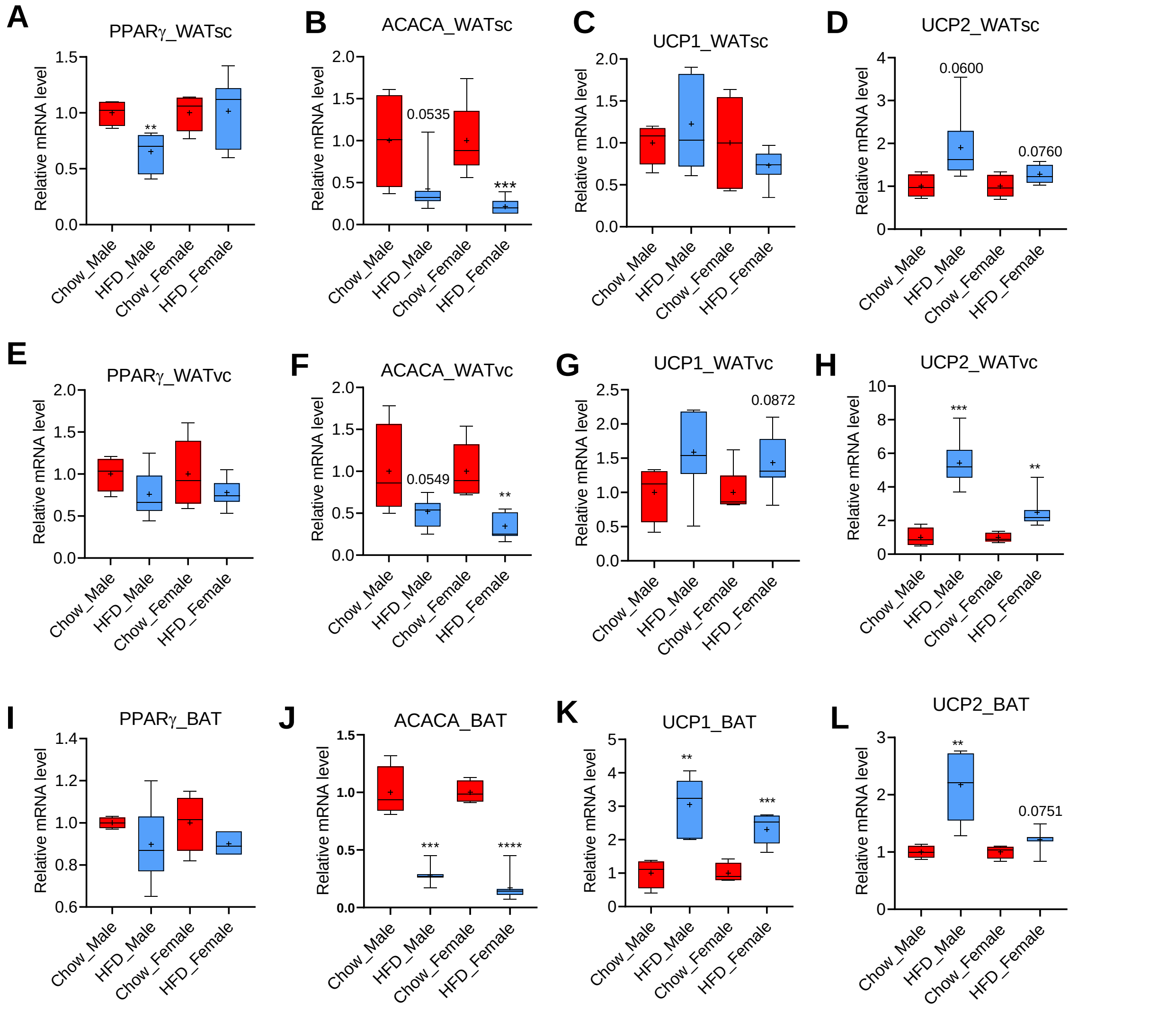

Supplemental Figure 4. Effect of HFD induced obesity on the expression of VDR target genes in subcutaneous white adipose tissue (WATsc), Visceral adipose tissue (WATvs), and the brown adipose tissue (BAT). The box-and-whisker plots indicate the minimum, the 25th percentile, the median, the 75th percentile, and the maximum. In addition, the mean is indicated with +. All data were analyzed with the two-tailed t-test.
